# Effects of Disinfectants on Larval Growth and Gut Microbial Communities of Black Soldier Fly Larvae

**DOI:** 10.3390/insects14030250

**Published:** 2023-03-02

**Authors:** Jianwei Hao, Shuang Liu, Zhixue Guo, Yan Zhang, Wuping Zhang, Chujun Li

**Affiliations:** 1 Department of Biological Science and Technology, Jinzhong University, Jinzhong 030600, China; 2 Institute of Loess Plateau, Shanxi University, Taiyuan 030006, China; 3 Xinzhou Livestock Development Center, Xinzhou 034000, China; 4 State Key Laboratory of Biocontrol, School of Life Science, Sun Yat-sen University, Guangzhou 510006, China

**Keywords:** black soldier fly larvae, pig manure, disinfectant, growth, gut microbiota

## Abstract

**Simple Summary:**

Swine manure is a significant source of agricultural organic waste, and in recent years, it has been shown that black soldier fly (BSF), *Hermetia illucens* (L.), (Diptera: Stratiomyidae), is effective at treating manures or compounds based on them. African swine fever virus (ASFV) infections have significantly modified the preventative procedure, including the disinfection of manure with various disinfectants, since 2018 when they first became a serious issue in the Chinese swine production industry. However, there is not any research discussing the influences of disinfectants in manures on black soldier fly larvae (BSFL). Therefore, this study examined the effects of the disinfectants contained in pig manure on the growth of BSFL and the reduction of waste. Additionally, the disinfectants might not only eliminate important pathogens but also alter the microbial composition of the gut of the larvae; thus, investigations of the intestinal bacterial communities of the BSFL-fed manures, which were mixed with various disinfectants, were conducted. These findings will be helpful in providing a better treatment of swine manures with BSFL.

**Abstract:**

The use of the black soldier fly has been demonstrated to be effective in the treatment of swine manure. Since the outbreaks of ASFV, prevention procedures, including manure disinfection, have changed dramatically. Glutaraldehyde (GA) and potassium peroxymonosulfate (PPMS) have been shown to be effective in the prevention of this pathogen and are thus widely used in the disinfection of swine manures, etc. However, research on the effects of disinfectants in manures on the growth of BSFL and gut microbiota is scarce. The goal of this study was to determine the effects of GA and PPMS on BSFL growth, manure reduction, and gut microbiota. In triplicate, 100 larvae were inoculated in 100 g of each type of manure compound (manure containing 1% GA treatment (GT1), manure containing 0.5% GA treatment (GT2), manure containing 1% PPMS treatment (PT1), manure containing 0.5% PPMS treatment (PT2), and manure without disinfectant (control)). After calculating the larval weight and waste reduction, the larval gut was extracted and used to determine the microbial composition. According to the results, the dry weights of the larvae fed PT1–2 (PT1: 86.7 ± 4.2 mg and PT2: 85.3 ± 1.3 mg) were significantly higher than those of the larvae fed GT1–2 (GT1: 72.5 ± 2.1 mg and GT2: 70 ± 2.8 mg) and the control (64.2 ± 5.8 mg). There was a 2.8–4.03% higher waste reduction in PT1–2 than in the control, and the waste reduction in GT1–2 was 7.17–7.87% lower than that in the control. In a gut microbiota analysis, two new genera (*Fluviicola* and *Fusobacterium*) were discovered in PT1–2 when compared to GT1–2 and the control. Furthermore, the disinfectants did not reduce the diversity of the microbial community; rather, Shannon indices revealed that the diversities of GT1–2 (GT1: 1.924 ± 0.015; GT2: 1.944 ± 0.016) and PT1 (1.861 ± 0.016) were higher than those of the control (1.738 ± 0.015). Finally, it was found that both disinfectants in swine manures at concentrations of 1% and 0.5% may be beneficial to the complexity and cooperation of BSFL gut microbiota, according to an analysis of microbial interactions.

## 1. Introduction

The continued increase in organic waste not only impacts human health but also threatens global ecosystems. The environmental problems caused by waste pollution are numerous, including water, air, and soil pollution [1]. Pathogens can also be a threat to human health along with waste pollution [2]. Surplus manure generated in areas with a high concentration of livestock, where animals are raised intensively, is a source of environmental pollution [3,4]. The pathogens contained in manure pollution could pose a threat to human and animal health [5]. Swine manure has been demonstrated to be a dissemination medium of various pathogens; thus, it is a target of disinfectant use. Following the outbreak of ASFV in China in August 2018 [6,7], the virus transmitted so fast that most of China’s provinces had been affected by April 2019 [8,9]. In addition, China is home to nearly half of the world’s pigs, and this virus resulted in significant economic losses. Thus, the use of extensive disinfectant sanitization on manure to control the transmission of pathogens has become an important procedure in swine production. However, there are few studies discussing the influences of disinfectants on waste treatment.

Insects are progressively gaining attention because of their ability to create protein, fat, and trace elements while consuming garbage, and in some food crisis areas, they have even supplanted livestock and meat as major sources of protein nutrition for humans [10]. Due to the black soldier fly’s varied feeding habits, high conversion rate, environmental friendliness, and inexpensive costs, it has recently received attention [11]. BSFL can be used to process various types of livestock manures [12,13]. It could perhaps offer a number of benefits at once. The physical, chemical, and biological features of manure can be altered by BSFL in one to two weeks, modifying the original moisture and nutrient levels in the process [14]. The weight of BSFL is a crucial index for the creation of biomass, particularly for further industrial use. This index may be influenced by a number of variables, including temperature, pH, the composition of the diet, and the density of the larvae [15]. Additionally, waste might contain some special influencing factors, such as insecticides, which are used for the pest control of food and might cause residues to remain in the waste [16]. Since the epidemic of ASF, swine manures might always be compounds that are mixed with disinfectants. According to our previous investigation, GA, sodium hydroxide, and PPMS are the main components of the treatment used to sanitize manure. In order to effectively disinfect the manure with a lower volume, a higher concentration of disinfectant is prepared in practice, and the prepared disinfectant is sprayed on the manure before the collection of manure. In the investigated farms, this process is typically repeated three times per day, and different disinfectants are available for alternative uses. Sodium hydroxide as a normal alkaline substance is used in various fields, including disinfectant and pH adjustment. Thus, it is hard to evaluate the influence of sodium hydroxide on larval growth simply based on its disinfection property. It has been found that GA has a disinfection effect not only for bacteria [17] but also for viral inactivation, such as coronavirus and ASF inactivation [18,19]. Regarding PPMS, its disinfectant effect has been found to be the same as that of GA [19]. It is worth noting that both PPMS and GA are widely used in pig farms, mainly for their high efficiency in ASF disinfection, at various concentrations (0.5% and 1% for PPMS; 0.1%, 0.5%, and 1% for GA) [19].

Thus, this study’s main objective was to conduct exploratory research on the effects of two disinfectants at various concentrations on the growth of BSFL. Our hypothesis was that the tested chemicals would impact BSFL growth when present in the feed substrate and that they could alter the gut microbial composition of the larvae, leading to functional changes. We selected growing-pig manure as the experimental substrate of BSFL because the growing period accounts for 64% (3.5 months/5.5 months) of the entire pig fattening period, resulting in the mass production of manure, as well as the excessive use of disinfectants. In addition, this manure type is suitable for BSFL growth according to our previous research [20]. The larval age (4–12), which is the most intriguing for commercially producing BSFL for feeding reasons, was the specific focus of the investigations. The results based on the representative disinfectants used in the farms can be used to identify the optimal aspects of high-grade insect products used to treat swine manures.

## 2. Materials and Methods

### 2.1. Acquisition of Pig Manures

Growing-pig manure was collected from a farm in Xinzhou, Shanxi province, People’s Republic of China. When the manure was collected, the growing pigs were 15 weeks old, and the manure samples were freshly excreted, which ensured that were was no exposure to disinfectants. The manure was placed in 10 L plastic bags, each weighing roughly 10 kg, and then it was delivered to the facilities at Jinzhong University. At the beginning of the experiment, the moisture content of the substrates was assessed using 10 g of each substrate and an electric oven SEB-3Y (Sanmai machinery Co., Ltd., Zhuhai, China) set to 65 °C for 24 h. A portable pH probe UB-7 (Denver instrument Co., Ltd., Arvada, CO, USA) was used to analyze the pH value. Before the experiment began, the manure-filled plastic bags were stored at −21 °C for preservation. The manure was given 24 h to defrost at room temperature before the experiment began [21]. The growing-pig manure had a 70% moisture content and a pH value of 5.6.

### 2.2. Experiment Design

Black soldier fly eggs were purchased from a company (Wuliang Biotechnical Co., Ltd., Guangzhou, China) and were incubated with wheat bran in a plastic box before being put in a climate-controlled incubator (Hengzi, Shanghai, China) to hatch. The hatching conditions were set at 28 °C and 75% humidity. According to a previous study [19], together with our investigation, different experiment groups were set up when the eggs hatched as follows: PT1–2, 100 4-day-old larvae cultured in 100 g of swine manure, which was mixed with PPMS (Macklin biochemical technology Co., Ltd., Shanghai, China) at concentrations of 1% and 0.5%; GT1–2, 100 4-day-old larvae cultured in 100 g of swine manure, which was mixed with GA (Macklin biochemical technology Co., Ltd., Shanghai, China) at concentrations of 1% and 0.5%; and control, 100 4-day-old larvae cultured in 100 g of swine manure without the addition of any disinfectants. Three replicates were used in each group, and each replicate was settled in a round plastic box (diameter: 9.8 cm; height: 10 cm). In addition, the 100 4-day-old larvae for each replicate were weighed to record the initial larval average weight and to ensure that there was no significant discrepancy within groups or between groups. By dividing the total dry weight of all the larvae from each replicate by the total number of 12-day-old larvae, after the larvae were removed from the media, the final larval average weight was calculated. The efficacy of the larvae in consuming and metabolizing the growing substrates was evaluated by weighing the total dry final biomass (feed) and the dry remaining substrates (residue). The ability of the larvae to reduce feeding substrates is shown by the waste reduction (WR) of the dry manures (DM). A greater ability to decrease the organic matter is indicated by higher values. The following is a computation of the equation [22]:

(1) $$\mathrm{Waste}\;\mathrm{reduction}\;\left(\%\;\mathrm{DM}\right)=\left(1-\frac{\mathrm{residue}\;\left(g\right)}{\mathrm{feed}\;\left(g\right)}\right)\times 100\%$$

### 2.3. The Gut Bacterial Community Analysis

The larval intestinal extraction method was similar to a previous study [23] as follows: (1) Put the insect on ice for three to five minutes, and then remove it. (2) To remove exterior pollutants, wipe the insect’s surface with 70% alcohol for 30 s, soak it in 0.25% sodium hypochlorite (Xilong scientific Co., Ltd., Shantou, China) for 1 min, and then rinse it three times with sterile water. (3) In a sterile environment, use sterilized fine-tipped forceps to cut open the abdomen of the insect, remove the entire intestine, and immediately rinse it twice with a 0.9% sterile NaCl solution. Then, the entire intestine can be taken out and placed in a microcentrifuge tube (1.5 mL). For each triplicate select about 10 BSF larvae whose guts were dissected as each larval sample (3 samples per treatment) and then store at −80 °C prior to DNA extraction. (4) A FastDNASpin Kit (MP biomedicals INC., CA, USA) can be used to extract the DNA.

Using an Illumina MiSeq PE300 platform (Majorbio Co., Ltd., Beijing, China), the V3–V4 regions of the 16 S rRNA genes from the intestinal DNA samples were sequenced and analyzed (Majorbio Co., Ltd., Beijing, China). The 338 F (GTACTCCTACGGGAGGCAGCA) and 806 R primer sets were utilized in the amplification reactions (GTGGACTACHVGGGTWTCTAAT). QIIME software was used to extract the high-quality sequences first, in accordance with previously described techniques. A 97% similarity was used to group the sequences into OTUs, which were then utilized to generate rarefaction curves, classify the sequences into operational taxonomic units (OTUs), and calculate diversity indices (Shannon and Simpson indices).

### 2.4. Data Analysis

Graphpad prism software (GraphPad Software INC., San Diego, CA, USA) was used to conduct a one-way ANOVA variance analysis and the least-significant difference (LSD) multiple comparison test for statistically significant differences among treatments (IBM Corp., Armonk, NY, USA). R version 3.6.1 was used to conduct NMDS, generate heatmaps, and determine the abundance of bacterial communities. We analyzed the correlations between the genera using pairwise Spearman correlations (r) in the psych package in R. The strong (r > 0.80 or r < −0.80) and significant (*p* < 0.05) correlations in the network analysis were used with Gephi v0.9.2. Other plots were produced using the Origin 2021 program.

## 3. Results

### 3.1. Performance of Larval Growth and Waste Reduction of Different Treatments

**Figure 1:**
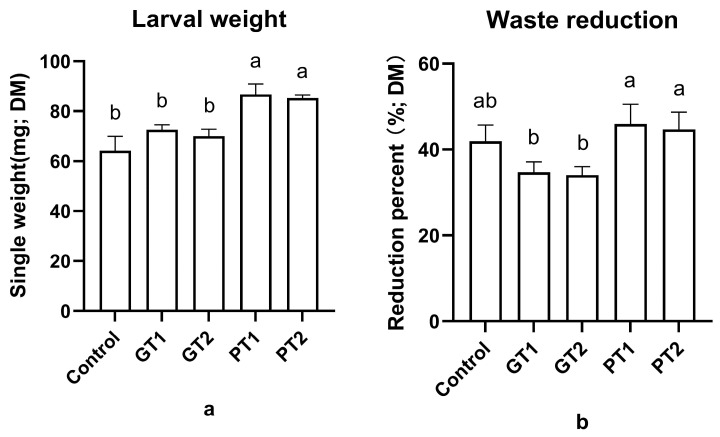
The larval weight and waste reduction of the pig manure with different concentrations of disinfectants: (**a**) larval weight of different groups; (**b**) waste reduction of different groups. Error bars represent standard deviation of triplicate. Columns marked by the same small letter do not significantly vary (*p* > 0.05).

A significant trial effect was found (F_4,10_ = 22.44; *p* < 0.01) between the different groups. There was no discernible difference in the larval weight between the control (64.2 ± 5.8 mg) and GT1 (72.5 ± 2.1 mg) or GT2 (70 ± 2.8 mg), whereas the larval weight results revealed a significant difference between the control and PT1 (86.7 ± 4.2 mg) and PT2 (85.3 ± 1.3 mg). In addition, the larval weights of both PT1 and PT2 were significantly higher than those of GT1 and GT2 (Figure 1a). It is worth noting that there was no significant difference in the larval weight between GT1 and GT2 or PT1 and PT2. The control had the lowest larval weight out of the different groups. Regarding the waste reduction of the different groups, a significant trial effect was also found (F_4,10_ = 7.635; *p <* 0.01). When compared to the control, there was a 2.8–4.03% higher waste reduction in PT1 and PT2; however, there were 7.17–7.87% lower values in GT1 and GT2 than the control (Figure 1b). Interestingly, a comparison of the results for the larval weight of the different groups revealed a discrepant trend in terms of waste reduction. The GT groups had a higher larval weight and a lower waste reduction than the control.

### 3.2. Effect of Disinfectants on the Communities of Intestinal Microbiota

**Figure 2:**
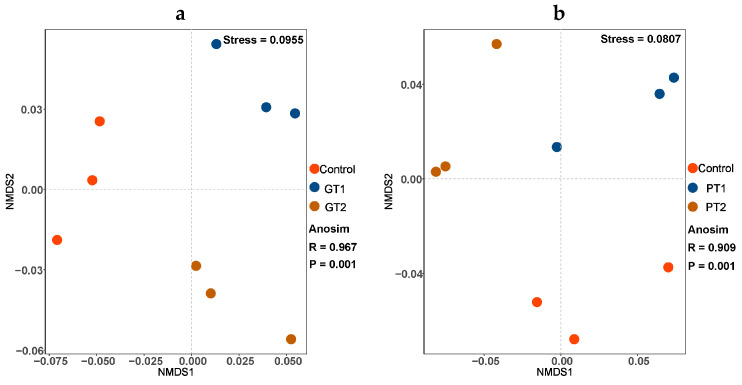
NMDS analysis based on Bray–Curtis distance, displaying the number of bacterial OTUs in BSFL gut shared among the control, GT1 and GT2 treatment groups (**a**), and the control, PT1 and PT2 treatment groups (**b**).

The larval gut samples of each treatment clustered well, according to the non-metric multidimensional scaling analysis (NMDS) (Figure 2). The points GT1 and GT2 correspond to the larval gut samples of the swine manures mixed with GA at two concentrations, and three separate groups are shown in Figure 2a. The larval gut samples of the swine manures mixed with PPMS at 1% or 0.5% or not mixed indicate the three separate bacterial communities that the larval gut microorganisms develop into (Figure 2b).

**Figure 3:**
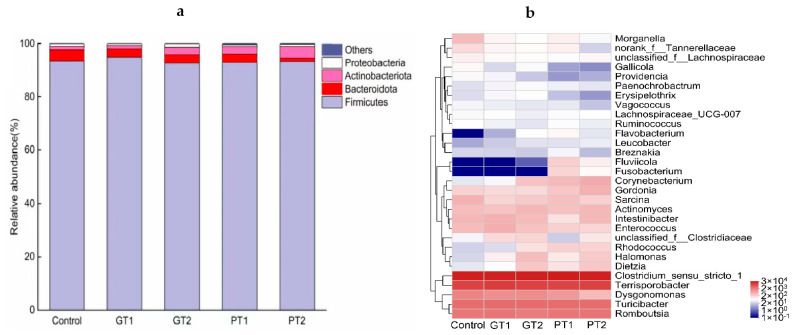
The relative abundances of the constitutive phyla (**a**), and heat maps of genera based on 16S rDNA (**b**) for the control and the GT1, GT2, PT1, and PT2 treatment groups. Bacterial OTUs counted below 1% of the total number of reads were categorized as other.

Firmicutes, Bacteroidota, Proteobacteria, and Actinobacteriota were the dominant phyla that made up more than 99% of all the microorganisms in the larval guts of all the treatment groups (Figure 3a). Firmicutes comprised 92.78−94.93% of the phylum of the different treatments. Additionally, for the GT1–2 and PT1 groups, the relative abundances of Bacteroidota and Proteobacteria were similar to those of the control; however, the abundances of Actinobacteriota in the GT2 (2.81%), PT1 (2.83%), and PT2 (4.26%) groups were two- to three-fold higher than those in the control (1.29%).

At the genus level, the bacterial community structure in the larval intestine was complex (Figure 3b). *Clostridium sensu stricto 1*, *Terrisporobacter*, *Turicibacter*, *Romboutsia*, and *Dysgonomonas* consistently made up more than 88% of the BSFL intestinal bacterial community across all the treatment groups, according to the main genera of the BSFL gut microorganisms. Another element was the considerable variation between PT1–2 and GT1–2 or the control in *Fluviicola* (which only existed in PT1–2), *Fusobacterium* (which only existed in PT1–2), and *Rhodococcus* (relative abundance: 2–10-fold higher in PT1–2 than in GT1–2 or the control). Additionally, the relative abundances of *Providencia*, *Gallicola*, *Sedimentibacter*, and *Enterococcus* were lower in PT1–2 than in GT1–2 and the control.

### 3.3. The Analysis of Microbial Community Diversity

**Figure 4:**
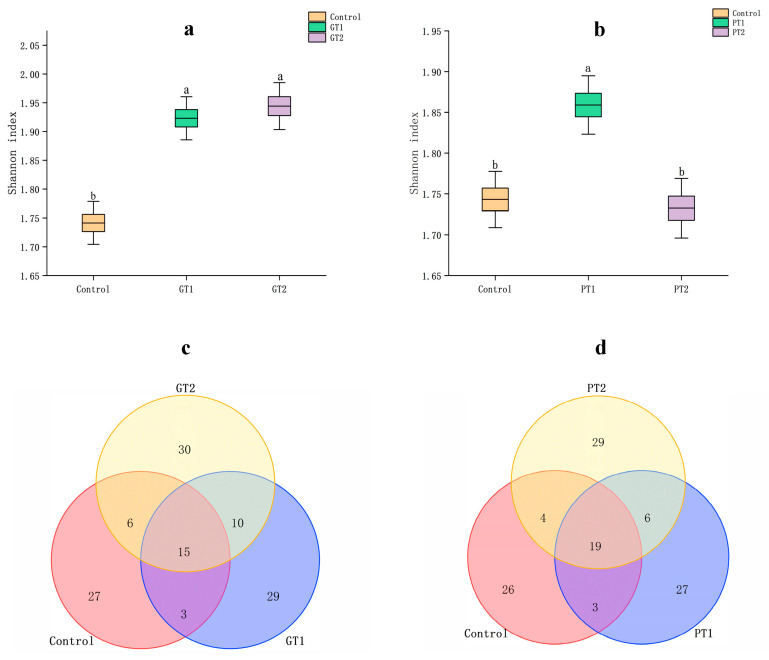
Bacterial community of Shannon diversity indices for the control, GT1 and GT2 treatment groups (**a**), and the control, PT1 and PT2 treatment groups (**b**). Venn diagrams of BSFL gut for the control, and GT1 and GT2 treatment groups (**c**) PT1 and PT2 treatment groups (**d**). Error bars represent standard deviation of triplicates. Columns marked by the same small letter do not significantly vary (*p* > 0.05).

Different samples had different Shannon diversity indices. The microbial diversity of the larval gut fed the GA-disinfected swine manures was relatively rich, as evidenced by the Shannon diversity indices of GT1–2 (GT1: 1.924 ± 0.015; GT2: 1.944 ± 0.016) being significantly greater than the indices of the control (1.738 ± 0.015) (Figure 4a). The indices of PT1–2 showed divergence in comparison to those of the control, with PT1’s index (1.861 ± 0.016) being higher and PT2’s index (1.736 ± 0.016) being lower than those of the control (Figure 4b). The Simpson indices revealed the same trends (Figure S1).

Venn diagrams showed that only 12.5% (15) and 16.7% (19) of the OTUs detected were shared between the control and the GA-exposed treatments and between the control and the PPMS-exposed treatments, respectively, and they provide additional evidence of the changes in the gut community structure that resulted from the exposure to both the disinfectants. Compared to the control, approximately 62 and 79 new genera were observed in the BSFL guts for the different disinfectant treatments (Figure 4c,d).

### 3.4. The Analysis of Microbial Networks of Different Groups

**Figure 5:**
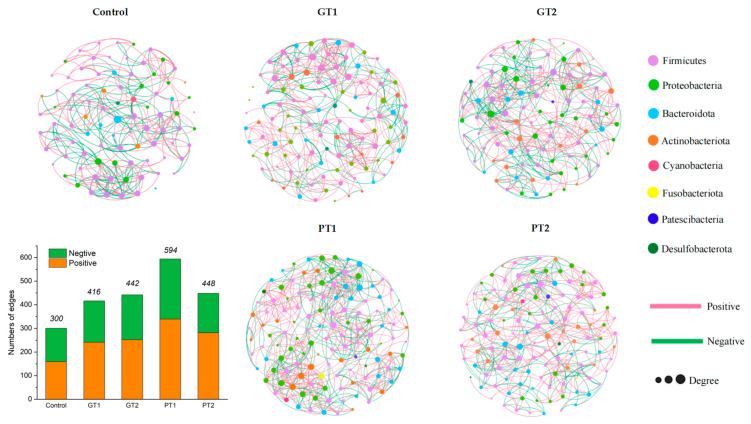
Effects of different disinfectant treatments on microbial networks. For easier visualization, nodes are colored according to the genera, and node size denotes the number of connections. The control represents the gut samples from the larvae fed pig manure without a disinfectant; GT1 and GT2 represent the gut samples from the larvae of GT1 and GT2; PT1 and PT2 represent the gut samples from the larvae of PT1 and PT2. The number of edges in the microbial networks that are both positive and negative is indicated. The values show the overall connections.

The microbial networks differed noticeably in the microcosms with and without the disinfectants, and variations in the same disinfectant at two concentrations were also noted (Figure 5). Generally, the control had the fewest nodes (88) in comparison to GT1–2 (GT1: 113 and GT2: 102) and PT1–2 (PT1: 127 and PT2: 123). There were more links in GT1–2 and PT1–2 than in the control (Figure 5). In the case of the GA treatment, GT1 had more interactions than GT2, whereas in the case of the PPMS treatment, PT1 had more interactions than PT2. In addition, PT1 had the most nodes and links compared to the other groups, and more nodes and links indicate a higher network complexity. The average degree of the control (6.307), which was lower than GT1–2 (GT1: 7.363 and GT2: 8.667) and PT1–2 (PT1: 9.354 and PT2: 7.285), was the lowest value of them all.

## 4. Discussion

The Chinese Ministry of Agriculture and Rural Affairs (MARA) reported 170 ASF outbreaks between August 2018 and May 2020, which led to a loss of 1.2 million pigs and a 40% decline in Chinese pork output [6]. At 4 °C, ASFV in pig manure might remain infectious for 8 days, whereas at 37 °C, it could last for 3–4 days. As a result, it should be understood that viable ASFV can spread through manures, especially within a herd [24]. PPMS and GA have been proven to be effective disinfectants in the elimination of ASFV [19], and according to our investigations, both have been widely, even excessively used in the sterilization of manures. Additionally, PPMS and GA can also inactivate other microbiota, such as bacteria. According to research, a 0.02% GA aqueous solution can effectively kill Gram-positive and Gram-negative bacteria, while a 2% solution can kill many kinds of microbial cells within 2 min, including *Staphylococcus aureus*, *Proteus vulgaris*, *Escherichia coli*, and *Pseudomonas aeruginosa* [25]. Further, while GA kills pathogenic bacteria, it could also have an impact on functional microorganisms in a wastewater biological treatment system and, subsequently, interfere with the performance of the wastewater treatment system [26]. PPMS is also effective in the disinfection of pathogens. When *Staphylococcus aureus*, *Pseudomonas aeruginosa*, and *Escherichia coli* were exposed to a 1% PPMS disinfectant solution for 22 s, the death rate reached 100%, according to research by Gasparini et al. [27]. Additionally, some studies have demonstrated that *Salmonella typhimurium* and *branching bacilli* are both effectively bactericidal due to PPMS [28,29]. Thus, we investigated the influences of the disinfectants in manures on larval growth, the waste reduction ratio, and the gut microbiota.

The results of the larval growth and waste reduction demonstrate that different disinfectants can impact the production of BSFL and waste reduction. The results of the weight of PT1–2 were significantly higher than those of the control and GT1–2, while GT1–2 did not significantly alter larval growth in comparison to that of the control larvae. In addition, PT1–2 had a higher ability of the larvae to reduce waste than GT1–2. It is worth noting that the results of the different concentrations of the same disinfectant are similar and do not differ significantly. PPMS is a peroxymonosulfate-based disinfectant that also acts as a potent oxidizer, targeting lignocellulosic components and promoting the breakdown of organic polymers. In addition, according to Wang et al., the peroxymonosulfate treatment of cotton straw and a cow manure mixture (MCC) resulted in a noticeable decrease in total solids and volatile solids. The rate of lignin removal could reach 30.8%, and organic materials were released throughout the process [30]. A previous study illustrated that cellulose and other types of fiber could slow larval growth, whereas non-fiber carbohydrates (NFCs) could be helpful in the growth of larval biomass [31]. Thus, it is possible that PPMS, which has the ability to decompose swine manure, will likewise have a positive impact on BSFL growth and the waste reduction ratio. Regarding GA treatments, the interaction of GA with amino groups in proteins and enzymes provides GA with disinfectant properties. Its two aldehyde groups also enable it to be used as a cross-linking agent, creating polymeric and nonpolymeric species while specifically targeting amino groups, which has biocidal effects [32]. We hypothesized that this mechanism could decrease protease activity, which is better for protein preservation, in comparison to that of the control, whereas protein cross-linking might affect how organic matter is digested. By catalyzing protein cross-linking, transglutaminase alters the viscosity, gelation, solubility, and water-holding capacity of peanut flour, according to Gharst et al. [33]. According to Duodu et al., protein cross-linking has the greatest impact on sorghum’s ability to digest its protein [34]. This is because protein has a significant impact on the development of BSFL [35]. Thus, the interaction of GA with protein may have an impact on waste reduction and larval growth. On the one hand, GA inhibition of protease activity could decrease protein degradation; on the other hand, cross-linking between GA and proteins in pig manure could reduce nutrient utilization. This could explain some of the characteristics of GA1–2, which showed similar larval weight to the control but significantly lower waste reduction than PT1–2.

The dominant phyla in the BSFL gut were Firmicutes, Bacteroidetes, Proteobacteria, and Actinobacteria (Figure 3a), which were also found to be the major phyla in the BSFL gut in an earlier study [36]. At the genus level (Figure 3b), a number of species, including *Morganella*, *Enterococcus*, *Providencia*, and *Dysgonomonas*, which have been proposed to be the core BSF gut microbiota members [2], were confirmed by a metagenomics study. It is remarkable that PT1–2 and GT1–2 had much lower *Morganella* and *Providencia* abundances than the control. Further, symbiotic bacteria serve crucial metabolic activities, such as food digestion, which are crucial for host survival and reproduction. According to a previous study, *Morganella morganii* and *Providencia* spp. together have the ability to produce urease, which results in the generation of significant amounts of biogenic amines, which could neutralize the gut’s acidic digestive fluids, preventing some bacterial species from being hydrolyzed [37]. Thus, the decline of *Morganella* and *Providencia in* GT1–2 and PT1–2 might have an impact on larval digestion and the gut microbial composition. In this study, high abundances of *Clostridium sensu stricto 1*, *Terrisporobacter*, *Turicibacter*, and *Romboutsia* were rarely detected in the BSFL gut; however, the opposite has been found in other studies. According to Wu et al., the gut of BSFL fed pig manure had much higher concentrations of the species of *Enterococcus*, *Clostridium sensu stricto 1*, and *Romboutsia*. They attributed this to the diet of the larvae [38]. Thus, the high-abundance genera in this study might have come from the pig manures. In comparison to the control and GT1–2, certain newly emerging taxa, including *Fluviicola* and *Fusobacterium*, were detected in PT1–2, and they likely also originated from the manures. In *Fluviicola* studies, it has been confirmed that both freshwater and wastewater contain this species, and this genus is characterized by strictly aerobic, Gram-negative, non-flagellated rods that move by gliding [39,40]. A study on this genus showed that it has the ability to incorporate nitrogen into the community [41]. Additionally, the research on *Fusobacterium* is always focused on oncobacterium [42], and there has been little discussion in the literature regarding this species in BSFL. Further, the relative abundance of *Rhodococcus* in PT1–2 was significantly higher than that of the control and GT1–2. This is noteworthy because this specie is highly adaptable to environmental stresses and is essential for environmental bioremediation [43]. An earlier study showed that adding *Rhodococcus rhodochrous* to the diet of BSF boosted its larval mass [44]. In this study, the higher relative abundance of *Rhodococcus* in PT1–2 might have also been helpful to the larval growth. Finally, compared with the control and PT1–2, in GT1–2, a higher abundance of *Clostridiaceae* was detected, which are common vertebrate decomposers [45]. According to previous research, the BSFL’s ability to convert organic waste can be enhanced by the addition of bacteria, and the BSFL gut microbes improve nutrition conversion from waste and expedite the bioconversion of organic waste. Microbial succession, according to Jiang et al., was significantly correlated with changes in metabolic functions and functional genes [46]. Furthermore, the gut microbiota and the enzymes they secrete do more than just break down harmful compounds and macromolecules; they also suppress infections, preparing the substrate for insects [47]. Research by Yu et al. into the gut microbes’ role in protein degradation by BSFL (germ-free or gnotobiotic) revealed the gnotobiotic treatment had a significantly higher protein reduction rate than germ-free treatment [48] Thus, the differences of microbiota between treatments in our study might also impact the process of the manure conversion with BSFL. However, the precise correlation between the gut microbiota and digestion needs to be researched in depth.

The Shannon value of PT1 is significantly higher than that of PT2 and the control, but for GT1 and GT2, they are both significantly higher than the control but similar to one another (Figure 4b), which might be caused by the various biocidal effects at different concentrations [19]. Contrary to our speculation, the diversity of the bacterial species in the BSFL gut of GT1–2 and PT1 was rather high when compared to that of the control (Figure 4a,b), indicating that GA and PPMS at certain concentrations (1% and 0.5% of GA; 1% of PPMS) did not lower microbial diversity. According to a previous study on the effects of chlorine disinfectant in freshwater on the intestinal microbial community of zebrafish, community diversity remained unaltered when compared to that of freshwater controls. The author also hypothesized that recovering intestinal microbial communities from dysbiosis may be a useful tactic to reduce the toxicity of a disinfectant [49]. Similar to this, our findings demonstrate that diversity was preserved in this experiment, even if the composition of the microbial community changed in response to the disinfectant. Co-occurrence networks can be used to analyze the complicated interactions between microorganisms, demonstrating the mathematical validity of microbial community aggregation [49]. As the main determinants of population structure and dynamics, these interactions are essential for the formation of microbial communities [50]. According to previous studies, a higher number of links and an average degree indicate that the networks are more complex, and networks with more complexity are typically more stable [51,52]. According to our findings, the numbers of links and the average degree of both GT1–2 and PT1–2 were higher than those of the control, which implies that both disinfectants may be advantageous to the complexity of the BSFL gut microbiota (Figure 5). In addition, the results also indicate an increase in the percentage of positive links in GT1–2 and PT1–2 in comparison to the control. Cooperation may be a key mode of interaction between bacteria in a relatively healthy gut [52]. Although digestion could be influenced by many factors, such as the composition of manures and dominant species, our findings suggest that BSFL fed swine manures treated with both disinfectants may have a more complex and cooperative microbial community at the very least.

## 5. Conclusions

This study indicates that GA and PPMS could impact growth and manure reduction when used at two concentrations that sterilize ASFV in swine manures. The biomass growth of BSFL was significantly accelerated by PPMS. GA decreased the waste reduction ratio but had no impact on the BSFL’s biomass growth. As disinfectants, GA and PPMS both have the potential to change the diversity, abundance, and interactions of gut microbes. Interestingly, the stability of the microbial community and the diversity of the larval gut microbiota did not decrease as a result of the disinfectants used; in fact, GT1–2 and PT1 even showed higher diversity indices than the control. It is worth noting that PT1 demonstrated the best performance not only in ASFV prevention but also in BSFL growth, and gut microbial complexity and cooperation. In conclusion, these findings are useful for the selection of disinfectants for pig manures used as larval diets, which will contribute to better manure treatment with BSFL.

## Data Availability

Data is available upon request from the corresponding author and pending agreement by co-authors.

## Supplementary Materials

The following supporting information can be downloaded at: https://www.mdpi.com/article/10.3390/insects14030250/s1, Figure S1. Bacterial community of Simpson diversity indices in BSFL gut for different treatments. Error bars represent standard deviation of triplicate. Columns marked by the same small letter do not vary significantly (*p* > 0.05).
