# Supplementary material for: Effects of Disinfectants on Larval Growth and Gut Microbial Communities of Black Soldier Fly Larvae
Source: Insects. 2023 Mar 2;14(3):250. doi: 10.3390/insects14030250 (PMC10056710; doi:10.3390/insects14030250)
Supplement: Supplementary file 1 [file insects-14-00250-s001.zip › insects-2206921-supplementary.pdf]

Supplementary Materials

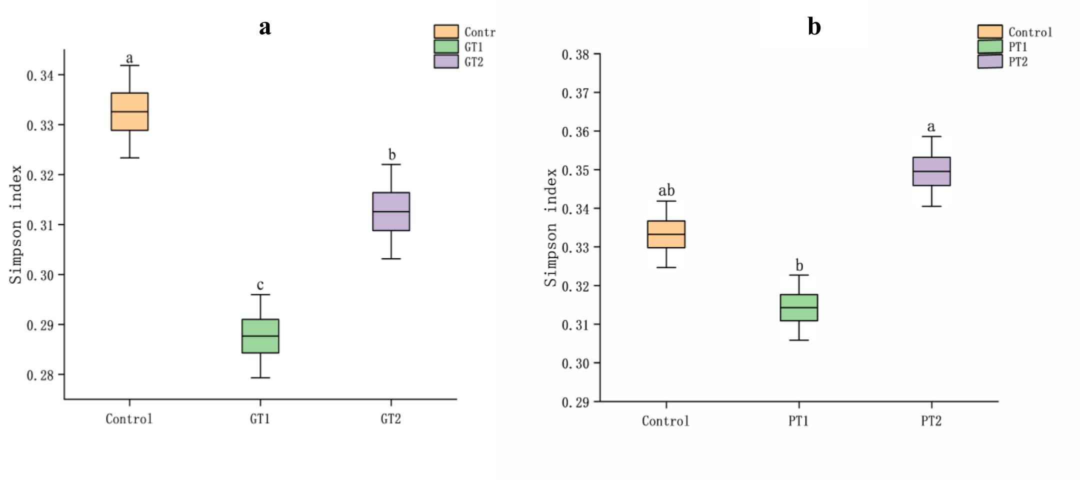

Figure S1. Bacterial community of Simpson diversity indices in BSFL gut for the control, GT1 and GT2 treatment groups (a), the control, PT1 and PT2 treatment groups (b). Error bars represent standard deviation of triplicate. Columns marked by the same small letter do not vary significantly ($p > 0.05$).
